# Inflammation – a blessing and a curse

**DOI:** 10.1007/s00709-026-02254-2

**Published:** 2026-08-12

**Authors:** Peter Nick

**Affiliations:** Joseph Gottlieb Kölreuter Institute für Plant Sciences, Karlsruhe Institute of Technology, Karlsruhe, Germany

The classic aspects of an inflammation (Celsus 35) – *rubor*, *calor*, *tumor*, *dolor* (redness, heat, swelling, pain) – might be symptoms of disease. However, they might as well be manifestations of defence against this disease. In fact, Hippokrates of Kos interpreted inflammations as manifestation of vigour, because the affected body musters heat beyond the normal degree, as mean to ward off disease (Hippokrates, ~ 410 BC). He understood the heat as active, but overshooting response to restore the temperature of the blood, because bile and phlegm had cooled it down before (Sticker 1929). Therefore, Hippokrates considered inflammation as part of πoνoς (*ponos*), the restorative effort of a healing body. However, if it continues unrestrained, the blessing can turn into a curse and the inflammation becomes a destructive aspect of παθoς (*pathos*). In fact, chronic inflammations have been recognised as central cause for numerous diseases that are otherwise difficult to be grouped into the same category (Chavda et al. 2024). On the level of chemistry, inflammation is linked with elevated levels of Reactive Oxygen Species (ROS). Again, these incompletely reduced derivatives of oxygen, bear two faces – if accumulating unchecked, they cause a plethora of cellular damage, if generated and dissipated in a timely manner, they are important signals coordinating the cellular response to stress. This dual nature of inflammation – activation and damage, or πoνoς (*ponos*) and παθoς (*pathos*) in the Hippokratian terminology – seems to date back to early evolution, because it can be detected in both animals and plants as is illustrated by several contributions to the current issue.

Osteoarthritis belongs to one of the most widespread chronic inflammations, where the cartilage in the joints is progressively breaking down. Therapeutic options are limited, the patients face a progressive loss of mobility and chronic pain, raising the interest into plant-based alternatives. In fact, ginseng extracts are used in Traditional Chinese Medicine to mitigate osteoarthritis. The bioactivity is ascribed to the triterpenoid glucoside ginsenoside Rh2. In their contribution to the current issue, Wang et al. (2026) investigate the molecular mode of action of this active compound using a cellular model (human chondrocytes, where an inflammatory response was evoked by lipopolysaccharides, mimicking a bacterial infection). The authors follow the accumulation of inflammation markers using a plethora of methods, such as cytokine quantification through ELISA, immunofluorescence of extracellular matrix components, or Western blot analysis of pyroptosis-related proteins to demonstrate that ginsenoside Rh2 can mitigate the inflammation, inhibiting the NF-κB signaling pathway, such that markers for pyroptosis as well as the secretion of pro-inflammatory cytokines is downmodulated. As a result, the inflammatory breakdown of the cartilage cells is avoided. The authors pursue the hypothesis that ginsenoside Rh2 achieves these effects, because it can act as a ligand for the receptor, normally addressed by the platelet-activating factor. They test and confirm this idea by molecular docking and thermal shift assays *in vitro.* To substantiate their prediction, they overexpress the receptor for the platelet-activating factor in the background of their chondrocyte model. In fact, the protective effect of ginsenoside Rh2 disappears upon overexpression of this receptor. Conversely, a knockdown of the receptor promotes the protective effect, consistent with the notion that ginsenoside Rh2, by binding to the receptor for the platelet-activating factor, can intercept inflammatory signalling. This mechanistic concept for the mode of action paves the way for testing the effect of this ginseng compound in higher-order models that approximate the situation in real patients.

Cardiovascular diseases are among the main death causes in industrialised societies worldwide, motivating the search for causes. High abundance of cholesterol in Low Density Lipid particles has been recognised as a factor often associated with this civilisation syndrome. However, the causative connection is far from well understood, while evidence for a chronic inflammation context is accumulating (for review see Tsoupras et al. 2018). Nevertheless, this discussion has stimulated a strong interest in low-cholesterol food. Especially, a meat-rich diet represents a major source for cholesterol intake, promoting the demand for low-cholesterol meat. In this context, admixture of soybean has become a common practice, especially in processed meat products. Soybean protein can be easily texturalised in a manner mimicking true meat and as a plant product, soybean contains less cholesterol. On the other hand, soybean can cause allergies, and also harbours phytoestrogens that can perturb hormonal balance and even contribute to certain tumours. Therefore, it is vital, that the consumers can arrive at an informed decision, whether they want to buy true meat or meat with admixtures of soybean. In their contribution to the current issue, Zaki et al. (2026) use a combination of microscopic diagnostics and metabolic analysis to investigate potential adulteration of meat products by soy proteins. Their data set is very extensive – more than 400 samples collected across supermarkets in Egypt, representing different types of meat products. Their histological images demonstrate convincingly, how meat and soybean protein can resemble each other in processed food samples. However, fluorescent dyes specific for plant components, such as cell wall or lignified vascular tissues, help to detect adulteration very efficiently and rapidly. They confirm their readout using ELISA and immunostaining with anti-soybean antisera, as well as by Scanning Electron Microscopy. In many cases, they even succeed to identify the source plant tissue in the commercial sample. The cholesterol levels measured by HPLC are much lower than to be expected for real meat samples. In conclusion, they can demonstrate massive admixture of soybean in a majority of the sampled products, most of them not declared at all, or, if just listed in a vague manner as “plant protein”. They calculate the economic losses resulting from the adulteration and arrive at actual costs that should be 80% lower than to be expected from the declaration as meat product. This meticulous study demonstrates, how it is possible, using traditional cell-biological methodology, to uncover food fraud very efficiently. The second message, beyond the startling outcome of extensive consumer deception which can even lead to health issues, is that the advocated focus on low-cholesterol food can backfire, if not accompanied by scientifically based check and balances.

While these two cases illustrate the curse of (chronic) inflammation, the contribution by Fábián (2026**)** adds a different facet, where oxidative burst acts as a blessing. The author investigates the question, why global climate change affects fertility and genetic stability of crop plants, scrutinising on meiosis as “Achilles’ Heel”. Climate change usually stands for heat stress, inducing DNA double-strand breaks, and microtubular breakdown with impacts on spindle formation and division axis. Drought stress, frequently acting in concert, perturbs post-meiotic pollen tube growth. However, climate change is not confined to those two abiotic stress forms. The need for artificial irrigation, in most cases with low quality water, increases the load of heavy metals causing oxidative burst impacting chromatin architecture resulting in aberrant chromosomes. Last, but not least, sudden cold snaps, due to blurred seasonality, disrupt the cytoskeleton, such that gametes remain unreduced. After reviewing the effect of climate-change associated abiotic stressors on meiosis, the authors funnel the manifold phenomena down to perturbed redox homeostasis, which is nothing else than a form of chronic inflammation. However, they also identify two positive aspects. On the one hand, a transient, restrained oxidative burst is required for meiosis to progress normally, a phenomenon also known from human ovaries (Tiwari and Chaube 2016). This “short-term” inflammation would correspond to the Hippokratian πoνoς (*ponos*). On the other hand, stress-induced meiotic aberrations will stimulate repair mechanisms such as increased recombination activity, leading to higher genetic diversity, which will promote evolutionary adaptation to the stressful condition. This path towards increased variation can be complemented by cytological mechanisms of genetic exchange, for instance by cytomixis, where chromatin can be exchanged through plasmodesmata between male meiocytes (Mursalimov et al. 2017). Eventually, perforated checkpoint control will increase genomic restructuring, such as polyploidy, which will expand ecological potency, because the individual can collect more than two allelic variants of essential proteins.

The contribution by de Souza et al. (2026) uncovers controlled inflammation as important driver for developmental processes. The motivation for their study was actually applied – working with the Amazonian *Piper aduncum*, a rare pepper accumulating dillapiole with interesting applications as biopesticides. As often in species that did not undergo a long history of domestication, the contents of this bioactive compound are variable, requiring a lengthy breeding process to arrive at an economic feasible production line. In plants, vegetative propagation provides a good alternative, but this species seems rather reluctant, leading to the question, how somatic embryogenesis could be stimulated. Motivated by this problem, the authors follow somatic embryogenesis *in vitro* using classic histology. They observe first cytological changes already three days after induction, including nuclear enlargement and formation of small meristematic cells along vascular bundles. These develop further into somatic embryos that soon form cotyledon-like organs and primary meristems that generate shoots. To understand, how this developmental switch generating new stem cells, is brought about, they investigate the machinery involved in redox balance. Interestingly, all of these enzymes exhibit a transient activity peak at the time of the reprogramming. At the same time, there is a peak in malondialdehyde, a stable indicator for lipoxygenation. Thus, a rapid, transient and constrained oxidative burst accompanies the transition from a somatic cell into a stem cell that is able to generate a new embryo.

Inflammation has originally been described by Hippokrates in the medicinal context and is, therefore, mostly discussed as a response specific for animals, maybe even for mammals. The undeniable fact that the responses during inflammation are very specific should not mask that some aspects central to inflammation seem to be evolutionarily ancient. Interestingly, the dual nature of inflammation (πoνoς, *ponos* and παθoς, *pathos*) is reflected in the dual nature of oxidative burst. If accumulating rapidly, transiently and in a restrained manner, reactive oxygen species are manifestation of vigour, activating stress adaptation or even developmental progression such as meiosis or somatic embryogenesis. If the come late, persistent and unchecked, the same reactive oxygen species are heralding pathological breakdown. What is valid for inflammation, holds true for oxidative burst as well – whether it acts as a blessing or as a curse, depends on timing.
